# Comparison of arterial wave intensity analysis by pressure–velocity and diameter–velocity methods in a virtual population of adult subjects

**DOI:** 10.1177/0954411920926094

**Published:** 2020-07-10

**Authors:** Ryan M Reavette, Spencer J Sherwin, Mengxing Tang, Peter D Weinberg

**Affiliations:** 1 Department of Bioengineering, Imperial College London, London, UK; 2 Department of Aeronautics, Imperial College London, London, UK

**Keywords:** Wave intensity analysis, pulse wave, systemic circulation, one-dimensional computational modelling, virtual population, ultrasound

## Abstract

Pressure–velocity-based analysis of arterial wave intensity gives clinically relevant information about the performance of the heart and vessels, but its utility is limited because accurate pressure measurements can only be obtained invasively. Diameter–velocity-based wave intensity can be obtained noninvasively using ultrasound; however, due to the nonlinear relationship between blood pressure and arterial diameter, the two wave intensities might give disparate clinical indications. To test the magnitude of the disagreement, we have generated an age-stratified virtual population to investigate how the two dominant nonlinearities, viscoelasticity and strain-stiffening, cause the two formulations to differ. We found strong agreement between the pressure–velocity and diameter–velocity methods, particularly for the systolic wave energy, the ratio between systolic and diastolic wave heights, and older subjects. The results are promising regarding the introduction of noninvasive wave intensities in the clinic.

## Introduction

Analysis of wave intensity (WI)^1^ is clinically useful, as arterial waves carry information about the performance of the heart and blood vessels,^2–6^ but its utility is restricted by its reliance upon measurements of blood pressure with high temporal resolution, which can only be measured invasively or estimated inaccurately from noninvasive methods. To obviate this restriction, Feng and Khir^7^ introduced a new formulation of WI that instead relies only upon diameter and velocity; both can be obtained from spatiotemporally coincident ultrasound images, but there is a lack of research on the efficacy of this noninvasive formulation as a surrogate for conventional WI.

Pressure and diameter are intrinsically related, and therefore the invasive pressure–velocity formulation and the noninvasive diameter–velocity formulation should be similar to some degree; however, the relationship itself is fundamentally nonlinear because of effects such as viscoelasticity and strain-stiffening.^8^ The objective of this study is to investigate how much these nonlinearities cause the two formulations to differ.

The study was conducted using 1D computational modelling of the arterial system, with simulations performed using the spectral/*hp*-element framework Nektar++,^9^ into which we incorporated a pressure–area relationship that models the aforementioned nonlinearities. This reduced-order modelling provides an effective means of studying arterial wave propagation, as it has the capacity to simulate complex networks with reasonable computational cost^10,11^ and has been validated against in-vitro^10,12^ and in-vivo^13–15^ data.

In this study, we have developed an age-stratified virtual population of adult subjects; age is a significant factor when investigating wave mechanics in the arterial system due to the stiffening of the arterial wall and increase of the arterial diameter over the adult lifespan. We present data for the common carotid, brachial and radial arteries and the thoracic aorta, as these are all accessible to ultrasound.

## Methods

### 1D computational formulation

Arterial networks may be approximated as a system of impermeable, compliant tubes having properties depending upon a single axial coordinate *x*. Applying mass and momentum conservation to an individual tube yields^11^

(1) $$\frac{\partial A}{\partial t}+\frac{\partial }{\partial x}\left(\mathrm{AU}\right)=0$$

(2) $$\frac{\partial U}{\partial t}+U\frac{\partial U}{\partial x}+\frac{1}{\rho }\frac{\partial P}{\partial x}=\frac{f}{\rho A}$$

where *A* is the cross-sectional area, *U* and *P* are, respectively, the cross-sectionally averaged velocity and pressure, *f* is the frictional force per unit length, and ρ=1060 kg/m^3^ is the density of blood.

Following Parker,^16^ we have supplemented the conservation equations with an exponential empirical law relating pulse wave velocity (PWV) to pressure

(3) $$c=c^{*}\exp\left[\frac{\alpha }{2\rho c^{{*}^{2}}}\left(P-P^{*}\right)\right]$$

where $c^{*}$ and $P^{*}$ are a convenient reference PWV and pressure, respectively, and α is a dimensionless free parameter which varies between each artery, is dependent on wall composition and describes the degree to which each artery exhibits strain-stiffening. Using the relationship

(4) $$c=\sqrt{\frac{A}{\rho }\frac{dP}{dA}}$$

allows us to express the law in the form P=P[A(x,t)] as

(5) $$P=P_{d}-\frac{\beta \sqrt{A_{d}}}{2\alpha }\ln\left[1-\alpha \ln\left(\frac{A}{A_{d}}\right)\right]+\frac{\Gamma }{\sqrt{A}}\frac{\partial A}{\partial t}$$

where $P_{d}$ and $A_{d}$ are the diastolic pressure and area, respectively, the rightmost term has been added to model viscoelastic effects, and β and Γ are the familiar stiffness and viscoelastic parameters from the commonly used Voigt-type viscoelastic tube law, respectively,^10^ and are defined as

(6) $$\beta =\frac{4\sqrt{\pi }\mathrm{Eh}}{3A_{d}}$$

(7) $$\Gamma =\frac{2\sqrt{\pi }\varphi h}{3A_{d}}$$

where *E* and φ are the elastic modulus and wall viscosity, respectively, and *h* is the arterial wall thickness. Notably, the choice α=0.5 results in this law closely mirroring the behaviour of the Voigt-type viscoelastic law; proof of this, along with other details about this tube law, can be found in Appendix 1.

**Figure 1.**
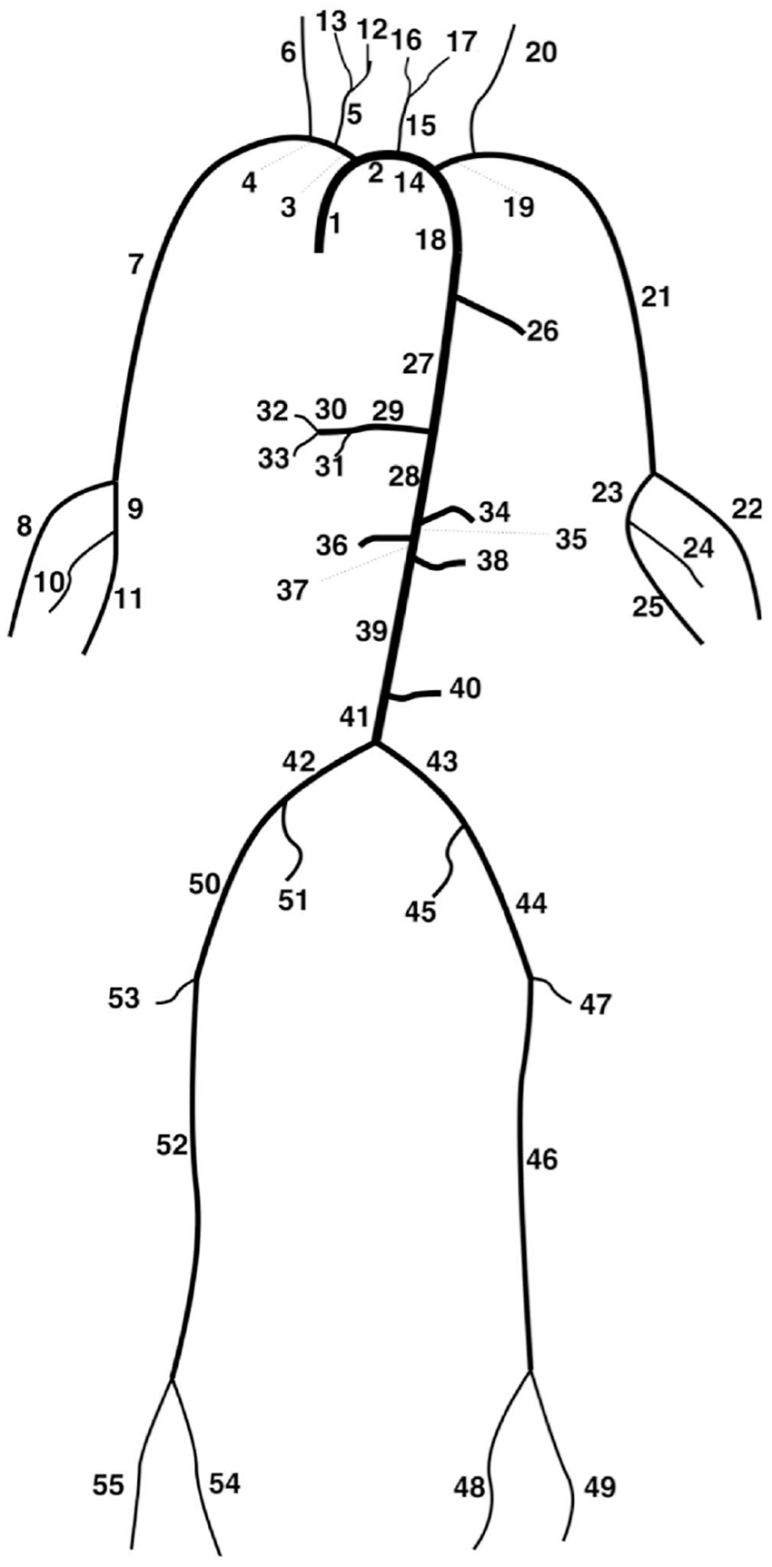
Depiction of the 55 artery network.^17^

Models used in the virtual population consisted of the 55 largest systemic arteries (Figure 1) modelled as linearly tapering vessels. Terminal vessels were coupled to RCR windkessel models.^8^ Simulations were conducted using the PulseWaveSolver utility of Nektar++,^9^ which uses a high-order discontinuous Galerkin method with a spectral/*hp*-element discretisation. Each simulation was conducted with a polynomial order of 3 and for sufficient cardiac cycles to ensure a periodic state was reached. For further details of the numerical method, the reader is referred to Sherwin et al.^11^

### Parameter estimation

#### Stiffness

Following Willemet et al.,^17^ β was estimated as

(8) $$\beta =\frac{2\rho }{\sqrt{A}}{\left(\frac{a_{c}}{D_{d}^{0.3}}\right)}^{2}$$

where $D_{d}$ is the diastolic diameter expressed in mm and $a_{c}$ is a PWV coefficient as introduced by Reymond et al.^18^ This method was chosen because clinical literature lacks information about arterial elastic moduli but does have data on PWV to which arterial stiffness is intrinsically related.

#### Wall thickness

Following Blanco et al.,^19^ the wall thickness *h* was approximated through the empirical law

(9) $$h=R\left(ae^{\mathrm{bR}}+ce^{\mathrm{dR}}\right)$$

where *R* is the lumen radius and with a=0.2802 , b=−5.053 cm^−1^, c=0.1324 and d=−0.1114 cm^−1^.

#### Strain-stiffening

Strain-stiffening results from the increased recruitment of collagen fibres and smooth muscle cells as arteries expand.^20,21^ The effects are larger in smaller, more muscular arteries as the arterial wall contains a larger proportion of these fibres; therefore, similar to Mynard and Smolich,^13^ we have assumed an inverse linear dependence of α on arterial diameter

(10) $$\alpha =\alpha_{0}+\frac{\alpha_{1}}{D}$$

where $\alpha_{0}=0.5$ such that large arteries exhibit behaviour that is close to elastic, and $\alpha_{1}=0.4$ cm^−1^ which gives good agreement with the data reported by Hisland and Anliker^22^ as presented by Parker,^16^ based on a typical diameter of the canine thoracic aorta.^23^ While approximate, this assumption gives appropriate results, with all arteries exhibiting a small but noticeable increase in elastic modulus with distending pressures in the physiological range,^24^ and with those with more collagenous and muscular walls showing the effect to a greater degree.

### Baseline model and the generation of virtual subjects

The baseline model used was taken from the work by Willemet et al.,^25^ with wall viscosities taken from the work by Alastruey et al.,^8^ as given in Table 1.

**Table 1.** Parameters of the baseline model.

Artery	Length *L* (cm)	Prescribed area $A_{d,\mathrm{in}}\to A_{d,\mathrm{out}}$ (cm^2^)	PWV coefficient, $a_{c}$ –	Wall viscosity, φ (kg/cm · s)	Peripheralresistance, *R* (kg/cm^4^s)	Peripheral compliance, *C* (cm^4^s/kg)
1. Ascending aorta	5.8	7.21 → 7.16	14.3	5	–	–
2. Aortic arch A	2.3	5.23 → 4.79	14.3	5	–	–
3. Brachiocephalic	3.9	3.40 → 2.69	14.3	10	–	–
4. R. subclavian	3.9	1.09 → 0.675	14.3	10	–	–
5. R. common carotid	10.8	1.00 → 0.270	14.3	60	–	–
6. R. vertebral	17.1	0.114 → 0.0651	15.6	60	45.1	0.00902
7. R. brachial	48.5	0.556 → 0.184	15.6	25	–	–
8. R. radial	27.0	0.114 → 0.0799	15.6	60	39.6	0.00987
9. R. ulnar A	7.7	0.114 → 0.0962	15.6	60	–	–
10. R. interosseous	9.1	0.0366 → 0.0269	15.6	60	632	0.00325
11. R. ulnar B	19.7	0.0850 → 0.0651	15.6	60	39.6	0.00769
12. R. internal carotid	20.5	0.271 → 0.153	15.6	60	18.8	0.0258
13. R. external carotid	18.7	0.0519 → 0.0186	15.6	60	104	0.0193
14. Aortic arch B	4.5	3.80 → 3.60	14.3	5	–	–
15. L. common carotid	16.0	0.785 → 0.198	14.3	60	–	–
16. L. internal carotid	20.5	0.154 → 0.0924	15.6	60	18.8	0.0189
17. L. external carotid	18.7	0.0305 → 0.0125	15.6	60	104	0.0173
18. Thoracic aorta A	6.0	3.33 → 2.99	14.3	5	–	–
19. L. subclavian	3.9	1.00 → 0.590	14.3	10	–	–
20. L. vertebral	17.0	0.114 → 0.0651	15.6	60	45.1	0.00902
21. L. brachial	48.5	0.546 → 0.184	15.6	25	–	–
22. L. radial	27.0	0.102 → 0.0651	15.6	60	39.6	0.00848
23. L. ulnar A	7.7	0.154 → 0.154	15.6	60	–	–
24. L. interosseous	9.1	0.0269 → 0.0269	15.6	60	632	0.00277
25. L. ulnar B	19.7	0.140 → 0.114	15.6	60	39.6	0.0130
26. Intercostals	9.2	1.33 → 0.751	14.3	5	60.0	0.104
27. Thoracic aorta B	12.0	2.27 → 1.39	14.3	5	–	–
28. Abdominal aorta A	6.1	1.25 → 1.25	14.3	5	–	–
29. Celiac A	2.3	0.506 → 0.395	14.3	5	–	–
30. Celiac B	2.3	0.225 → 0.200	14.3	5	–	–
31. Hepatic	7.6	0.243 → 0.161	15.6	25	27.2	0.0205
32. Gastric	8.2	0.0850 → 0.0750	15.6	60	40.6	0.00821
33. Splenic	7.2	0.147 → 0.127	15.6	60	17.4	0.0140
34. Superior mesenteric	6.8	0.519 → 0.420	14.3	10	6.98	0.0481
35. Abdominal aorta B	2.3	1.09 → 1.06	14.3	5	–	–
36. L. renal	3.7	0.225 → 0.225	14.3	25	8.48	0.0231
37. Abdominal aorta C	2.3	1.15 → 1.15	14.3	5	–	–
38. R. renal	3.7	0.225 → 0.225	14.3	25	8.48	0.0231
39. Abdominal aorta D	12.2	1.11 → 1.00	14.3	5	–	–
40. Inferior mesenteric	5.8	0.184 → 0.0844	15.6	25	51.6	0.0133
41. Abdominal aorta E	2.3	0.968 → 0.899	14.3	5	–	–
42. L. common iliac	6.8	0.519 → 0.407	18.0	10	–	–
43. R. common iliac	6.8	0.519 → 0.407	18.0	10	–	–
44. L. external iliac	16.6	0.341 → 0.310	18.0	25	–	–
45. R. internal iliac	5.8	0.133 → 0.133	19.7	60	59.6	0.0137
46. L. femoral	50.9	0.225 → 0.120	19.7	60	–	–
47. L. deep femoral	14.5	0.133 → 0.114	19.7	60	35.8	0.0127
48. L. posterior tibial	36.9	0.0799 → 0.0651	19.7	60	106	0.00743
49. L. anterior tibial	39.8	0.0564 → 0.0441	19.7	60	106	0.00513
50. R. external iliac	16.6	0.341 → 0.310	18.0	25	–	–
51. R. internal iliac	5.8	0.133 → 0.133	19.7	60	59.6	0.00137
52. R. femoral	50.9	0.225 → 0.120	19.7	60	–	–
53. R. deep femoral	14.5	0.133 → 0.114	19.7	60	35.8	0.0127
54. R. posterior tibial	36.9	0.0799 → 0.0651	19.7	60	106	0.00743
55. R. anterior tibial	39.8	0.0564 → 0.0441	19.7	60	106	0.00513

PWV: pulse wave velocity.

In addition to these, $P_{d}$ and $P_{\mathrm{out}}$ were 10 kPa (75 mmHg) and 1.33 kPa (10 mmHg), respectively.

Three-hundred subjects were generated, with 50 in each age group. For each subject, model parameters were scaled with randomly generated multipliers that lie in the ranges given in Table 2, which was based on a similar table given in the work by Willemet et al.^25^ The ends of each range were chosen such that there was the largest possible variation with no two age classes overlapping and with the middle of each class the same as the values given in the original table.

**Table 2.** Multipliers for the parameters that were known to vary between age groups.

Parameter	Multiplier					
	Age group (year)					
	20–29	30–39	40–49	50–59	60–69	70–79
Elastic arteries PWV $\left(c_{\mathrm{elas}}\right)$	0.7–0.9	0.9–1.1	1.15–1.45	1.45–1.75	1.75–2.05	2.075–2.425
Muscular arteries PWV $\left(c_{\mathrm{musc}}\right)$	0.7–0.9	0.925–1.075		1.075–1.225		1.225–1.375
Elastic arteries diameter $\left(D_{\mathrm{elas}}\right)$	0.9–1			1.1–1.3		1.3–1.5
Muscular arteries diameter $\left(D_{\mathrm{musc}}\right)$	0.9–1			1.105–1.305		

PWV: pulse wave velocity.

Arteries were categorised based on structure: 1–5, 14, 15, 18, 19, 26–30, 35–39 and 41 as elastic and the rest muscular.

**Figure 2.**
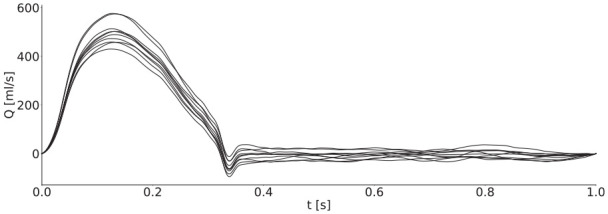
Ten example inflow waveforms with periods scaled to match to allow easier comparison. Peak volume fluxes were between 400 mL/s and 600 mL/s, and stroke volumes were between around 60–110 mL, which is within the physiological range for healthy adults.^27^

For the inflow waveform, we took that given for the thoracic aorta by Boileau et al.^26^ and modified its Fourier harmonic representation to induce physiological variation (Figure 2). In addition to this, we varied the heart rate between 60 and 80 beats per minute (with systolic time intervals scaled to match^28^) and the stroke volume with a multiplier of 0.8–1.2.

For all groups, the total peripheral resistance *R*, total peripheral compliance *C*, artery lengths $L_{i}$ , strain-stiffening parameters $\alpha_{i}$ , wall viscosities $\varphi_{i}$ , diastolic pressure $P_{d}$ and outflow pressure to the venous system $P_{\mathrm{out}}$ were varied with multipliers of 0.9–1.1. Doing so provided additional variation between subjects without inducing significant deviation from published values.

As a result of these variations, the stiffness parameter β and the viscoelastic parameter Γ calculated through equations (6) and (7) were changed through a combination of the formula for estimating wall thickness and the multipliers for diameter, PWV and wall viscosity.

### WI analysis

WIs were calculated using a sampling period Δt of 1 ms. Conventional WI magnitudes depend on the sampling period; throughout this work, they were divided by $(\Delta t{)}^{2}$ to remove this dependence

(11) $$dI=\frac{dPdU}{{\left(\Delta t\right)}^{2}},{\;}_{n}dI=\frac{dDdU}{{\left(\Delta t\right)}^{2}}$$

This has the disadvantage that neither of the WI units have an apparent physical meaning; however, it allows for simpler comparison between the data provided in this article and other measured WIs available in the literature. To ensure the same scale of magnitude for separated waves, the formulae taken from the work by Khir et al.^29^ and Feng and Khir^7^ were also divided by the same factor

(12) $$dI_{\pm }=\pm \frac{1}{{\left(\Delta t\right)}^{2}}\frac{1}{4\rho c}(dP\pm \rho cdU{)}^{2}$$

(13) dIn±=±1(Δt)2c2D(dD±D2cdU)2

Note that these formulae include the PWV, *c*; it is theoretically calculable from equation (4), but this requires both pressure and diameter. In practice, the arterial variables are measured in other pairs: pressure and velocity invasively with a catheter or diameter and velocity noninvasively with ultrasound or magnetic resonance imaging (MRI). Consequently, in the clinic, the PWV must be determined through means such as the *PU*-loop^29^ or the lnDU -loop.^7^ Willemet et al.^30^ found that these methods give estimates that can deviate significantly from the true value and from each other, particularly when close to reflection sites as in the case of the carotid and radial arteries. Errors from these loop methods will affect WI metrics involving separated waves and hence may cause further disparity between the two WI methods. To elucidate the size of this effect, separated waves were calculated with both the true PWV and the PWV derived from the respective loop method.

**Figure 3.**
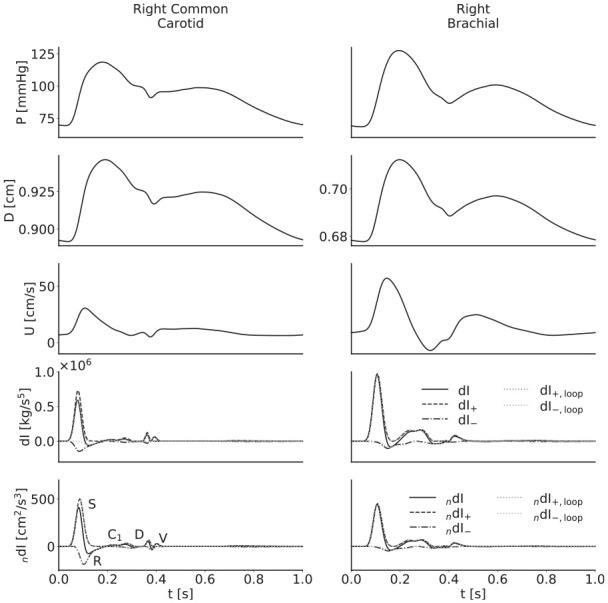
From top to bottom, pressure, diameter, velocity, invasive wave intensity and noninvasive wave intensity waveforms for the baseline model in the right common carotid (left) and right brachial (right) arteries. The S wave results from systolic contraction; the R wave is a reflection from a distal site of the S wave; the D wave results from diastolic relaxation; and the V wave results from the closure of the aortic valve, which is in turn responsible for the incisura. The origin of the C_1_ wave remains to be established definitively, though Curtis et al.^2^ have attributed it to reflections which travel back through the aorta and into the suprathoracic region; this wave presents differently in the carotid and the brachial arteries.

When discussing the waves, the prefix _n_ refers to the noninvasive method, and invasive and noninvasive WI refer to those calculated with the theoretical PWV, unless otherwise stated. All labels correspond to the separated components as defined in equations (12) and (13) and shown in Figure 3.

#### Comparison metrics

Six metrics were used:

1. *Pearson correlation coefficients* between the d*I* and dIn waveforms.
2. *Reflection coefficient* – the ratio of the magnitudes of the R wave and S wave.
3. *Systolic wave energy ratio* – the integral of the S wave divided by the total integral of the d $I_{+}$ or dIn+ waveform over one period.
4. *Peak ratio* – the ratio of the magnitudes of the D wave and S wave.
5. *Start delay* – the time difference between the S wave and the D wave first hitting 5% of their respective peaks.
6. *Peak delay* – the time difference between the arrivals of the peaks of the S wave and R wave, relative to the opening of the valve.

### Filter criteria

Following Willemet et al.,^25^ in order to ensure simulated subjects had physiological waveforms, a number of filter criteria were applied: diastolic and systolic blood pressure at the brachial artery were above 40 mmHg and below 200 mmHg, respectively; pulse pressure at the brachial artery was between 25 mmHg and 100 mmHg and the reflection coefficient at the aortic bifurcation was between −0.3 and 0.3. The reflection coefficient was calculated as

(14) $$R_{a}=\frac{Y^{a}-Y^{b}-Y^{c}}{Y^{a}+Y^{b}+Y^{c}},\;\mathrm{where}\;Y^{i}=\frac{\rho c_{i}}{A_{i}}$$

for the time-averaged cross-sectional area and PWV. Whenever subjects did not pass all filter criteria, replacements were generated.

## Results

### Waveforms

WI plots for the right common carotid and right brachial arteries of the baseline model, with labelled waves, are given in Figure 3. In these examples, the separated waves calculated with the loop-derived PWV match those calculated with the theoretical PWV well, except in the case of the invasive WI in the carotid, where instead they are close to the unseparated WI. The use of the incorrect PWV is evident from the presence of small but noticeable self-cancelling waves towards the end of the cardiac cycle; there is no physiological reason for such waves to exist, but they are the only way the separation theory can reconcile the error in the PWV to give a net WI of zero.

**Figure 4.**
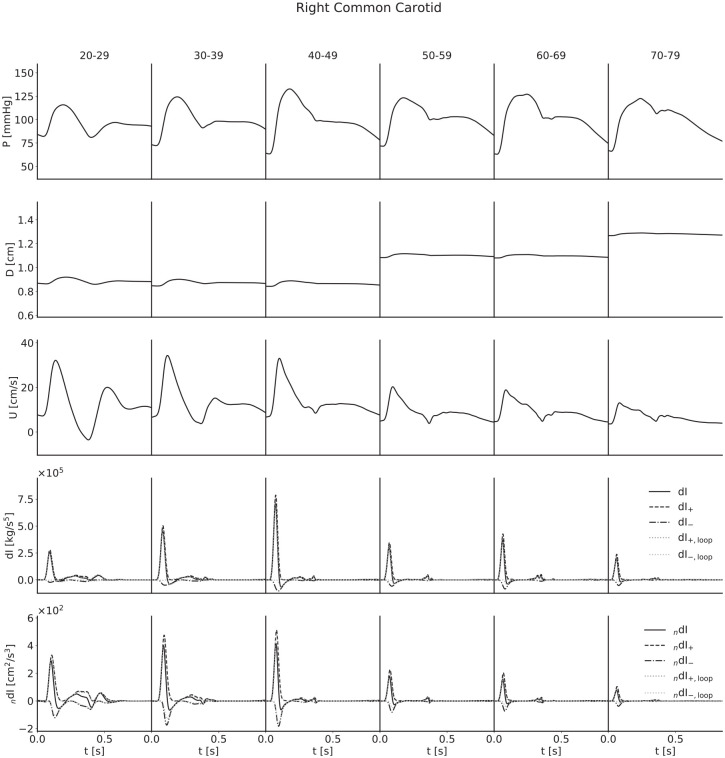
Ensemble-averaged waveforms for the right common carotid artery.

**Figure 5.**
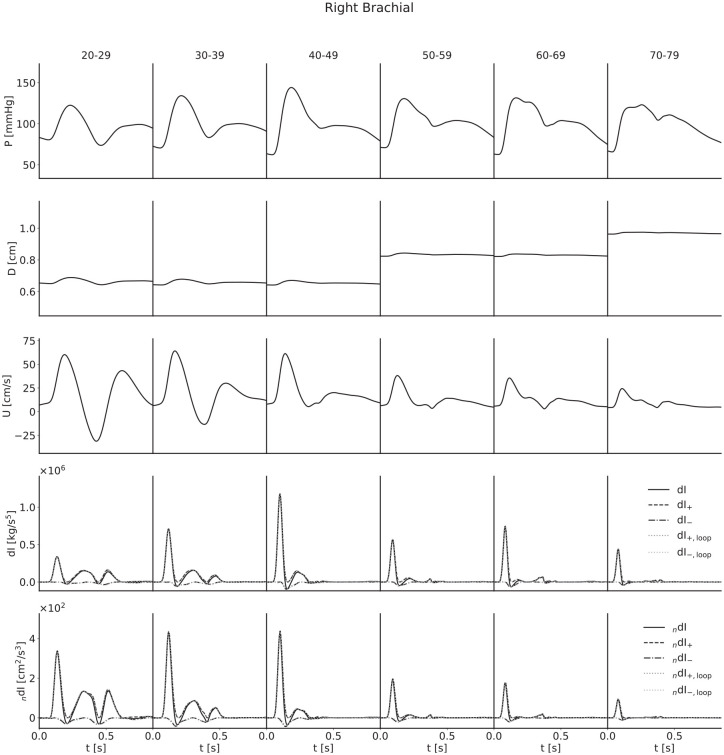
Ensemble-averaged waveforms for the right brachial artery. The wave intensity waveforms of the youngest subjects present a large C_1_ wave; in this case, it is a forward travelling decompression wave which decelerates flow and results in a relatively large backflow.

**Figure 6.**
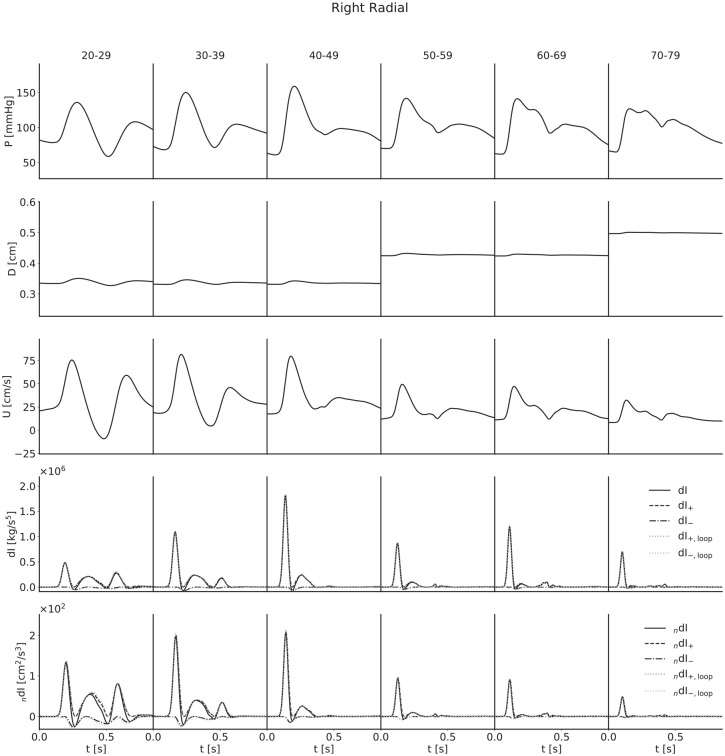
Ensemble-averaged waveforms for the right radial artery. The pulse wave velocity from the lnDU -loop introduces slight errors when separating forward and backward waves with the noninvasive formulation in the youngest age group.

**Figure 7.**
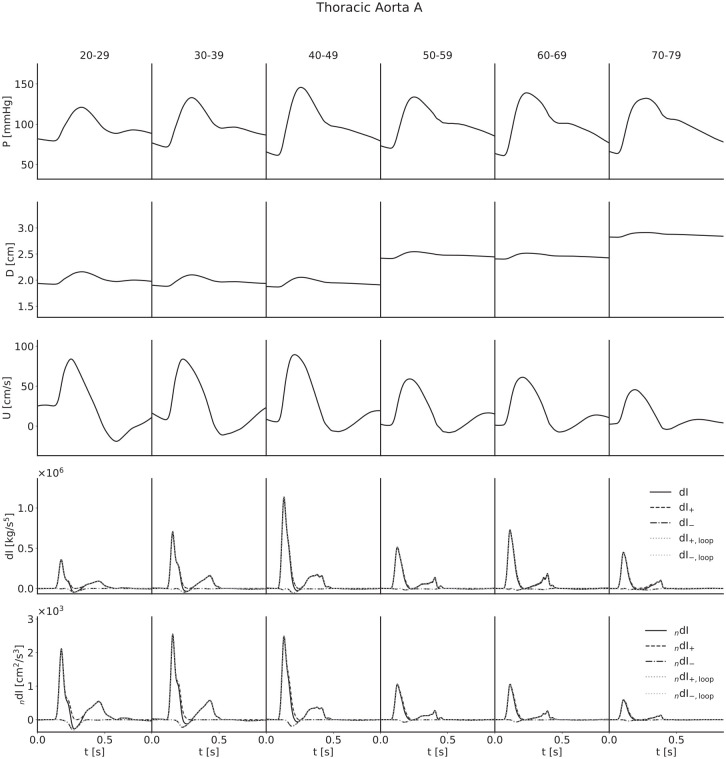
Ensemble-averaged waveforms for the thoracic aorta.

For each artery, age-stratified waveforms were ensemble-averaged across subjects; they are presented in Figures 4–7. The waveforms for the radial artery are similar in nature to the brachial, but they have nevertheless been included to show the effects of the loop methods on wave separation.

For both pressure and velocity, all ensemble-averaged waveforms lie within the physiological range and match those reported in the literature.^8,13,31–33^ Furthermore, for pressure, the progression with age is as expected: as age increases, the arteries stiffen and PWV increases, leading to both an increased pulse pressure (for age groups with similar diameters) and a narrowing of the dicrotic notch as the reflection of the initial pressure wave arrives earlier. The diameter waveforms are of similar shape to those of pressure and have been included with the same scale to show the magnitudinal differences between age groups as a result of the multipliers given in Table 2. For flow velocity, again the progression with age is as expected: as age increases, the arteries increase in diameter and so mass conservation dictates the velocities decrease, in addition to arterial stiffening reducing the magnitude of deviations from the mean velocity.

The invasive WI waveforms also match those available in the literature.^1,2,13,34^ S and R waves are present in all arteries, but it is important to note that an overall smoothing of the waveforms as a consequence of the ensemble averaging has reduced the magnitude of the D and V waves in the carotid, brachial and radial arteries. In general, we would expect larger presentations of the D wave when considering individual subjects, and we would expect to observe the V wave, which for a large number of age groups has been lost completely. The thoracic aorta has not experienced the damping of the D wave to the same degree, indicating that waveforms are largely similar between subjects. The V wave is not observable in the thoracic aorta, but this is expected as the incisura progressively dampens with distance along the aorta, so this is not a consequence of the ensemble averaging.

For all arteries, there is an increase in the magnitude of the invasive WI between the 20–29 and 40–49 year age groups and the 50–59 and 60–69 year age groups, resulting from increased wall stiffness: as the arteries become more difficult to distend, the same increase in volume will cause a faster increase in pressure, while mass conservation will dictate a faster rate of increase of velocity. This effect is not seen in the noninvasive WI, however; the magnitude is approximately constant between the 30–39 and 40–49 year age groups for the carotid, decreases between the same age groups for the brachial and radial arteries and thoracic aorta and decreases between the 50–59 and 60–69 year age groups for all four arteries. We attribute this to strain-stiffening: the sharper increase in pressure does not coincide with an equal increase in diameter, as the arteries grow more difficult to distend in these conditions. In support of this, simulations conducted in the absence of the nonlinearity resulted in similar increases in invasive and noninvasive WI.

The early increase in magnitude of the peak WI is counteracted by the increase in diameter between the 40–49 and 50–59 year age groups and the 60–69 and 70–79 year age groups, resulting in a net decrease with age for all arteries.

The C_1_ wave also shows disparities between age groups. This wave is most significant in the youngest subjects and diminished in the older groups. This is seemingly counter-intuitive, as viscoelasticity decreases with age, and viscoelastic damping is expected to reduce the influence of amalgamated reflections to which this wave has been attributed;^2^ instead, it would appear that the fact that younger arteries are more distensible results in a smaller level of damping overall.

The loop methods separate the waves well for all ages in both the brachial artery and thoracic aorta, but the lnDU -loop performs poorly in the radial artery of the youngest subjects, and both methods perform poorly for all ages in the common carotid, particularly for the invasive WI, where the *PU*-loop gives a PWV that fails to provide any meaningful separation. The noninvasive lnDU -loop fares slightly better but is responsible for a decrease in magnitude of both the S and R waves in multiple cases.

### Statistical analysis

**Figure 8.**
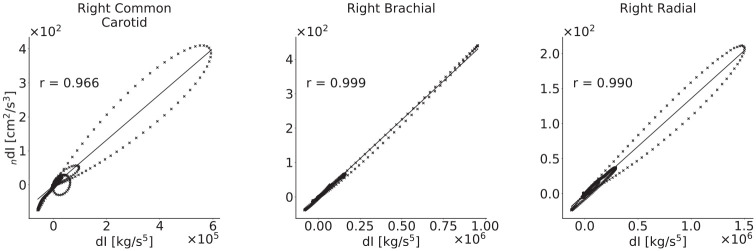
Correlation coefficients between the d*I* and _*n*_d*I* waveforms for three arteries in the baseline model. All are very close to 1, indicating that the two wave intensities are similar as expected. The greatest variation occurs in the carotid and radial arteries; this is indicative of the significant effect of viscoelasticity, as these arteries were modelled to have the largest wall viscosities.

Pressure–diameter graphs for three arteries of the baseline model, with correlation coefficients, are given in Figure 8; these illustrate the nonlinear relationship between the variables.

**Figure 9.**
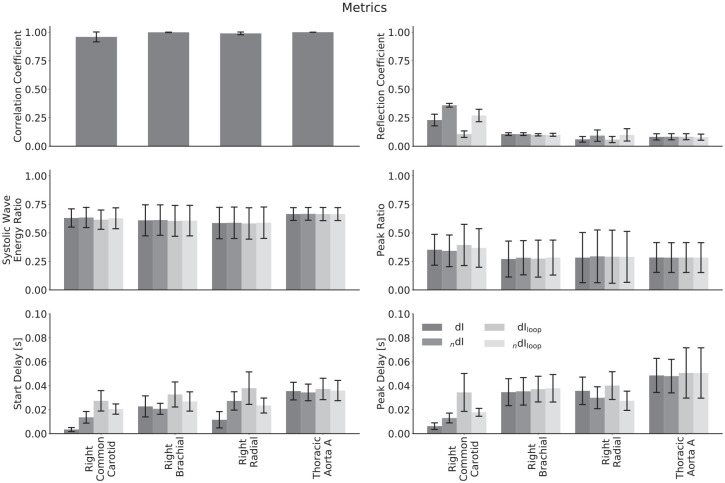
Comparison of metrics derived from invasive and noninvasive wave intensity analysis averaged across all subjects for different arteries. The biggest differences are for the reflection coefficients and delays in the common carotid and radial arteries. The systolic wave energy ratio and peak ratio show excellent agreement for all arteries.

**Figure 10.**
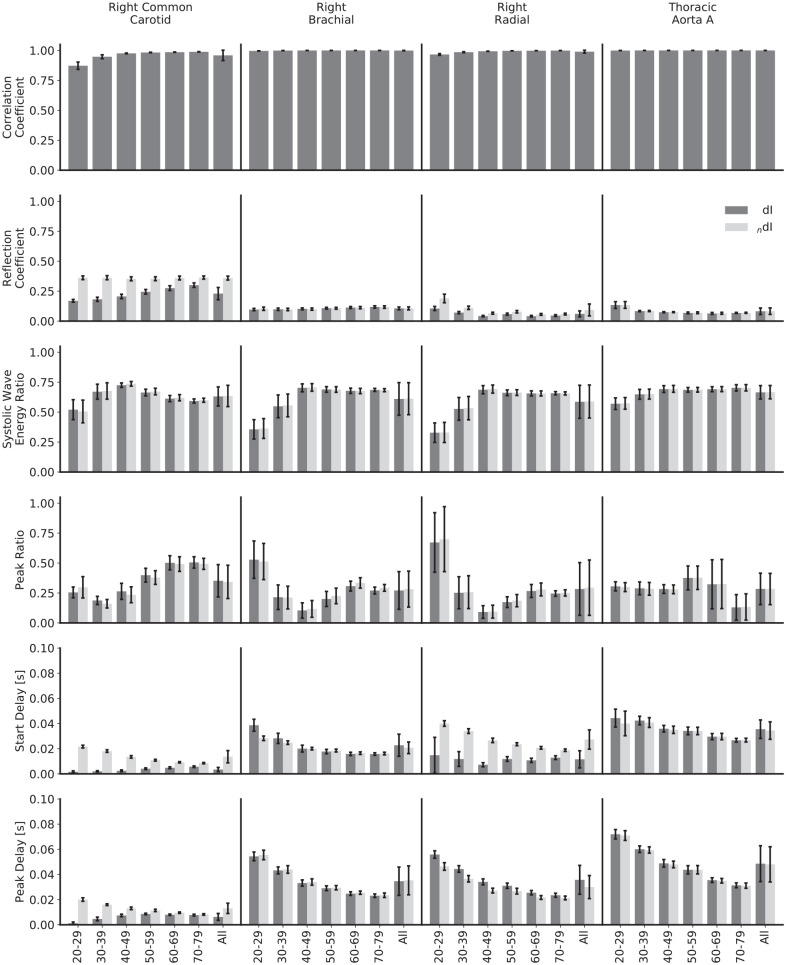
Comparison of waveforms and metrics for invasive and noninvasive wave intensity analysis, calculated with ideal pulse wave velocities and stratified by age and artery. The biggest differences between the two formulations are for the reflection coefficients and delays in the common carotid and for the start delays in the radial: in all these cases, the two WIs show different trends as age increases.

**Figure 11.**
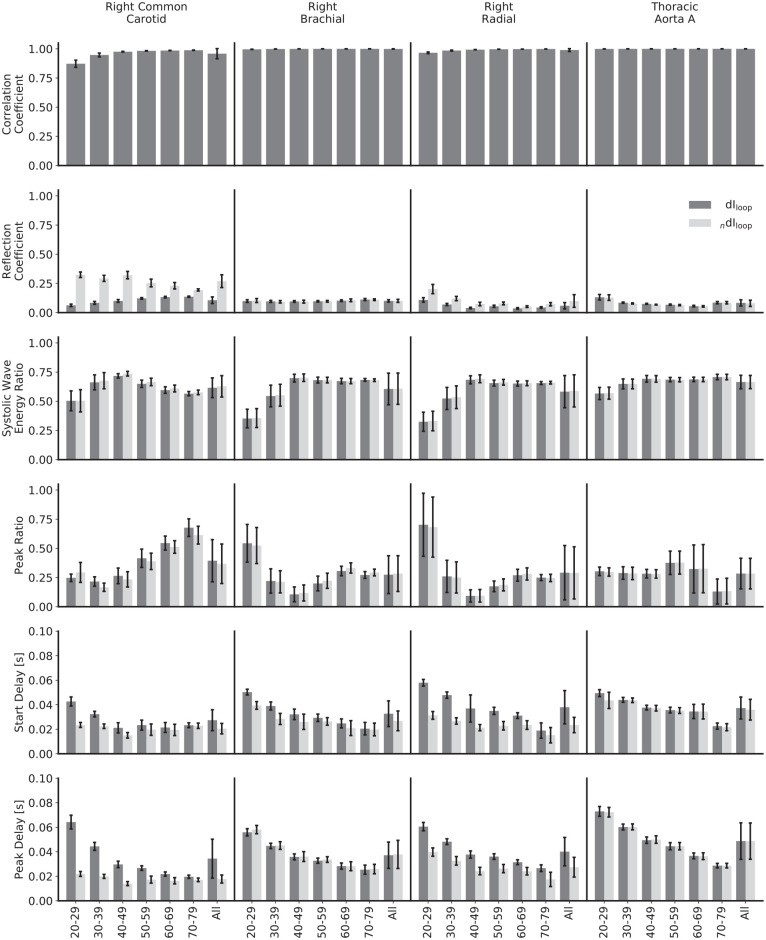
Comparison of waveforms and metrics for invasive and noninvasive wave intensity analysis, calculated with loop-derived pulse wave velocities and stratified by age and artery.

The mean values for the comparison metrics across all subjects are presented in Figure 9, the same metrics stratified by age group and calculated using the theoretical PWV in Figure 10 and the respective loop-derived PWV in Figure 11. Standard deviations have been used to display the errors.

The mean correlation coefficients for all arteries are close to 1, indicating that the modelled nonlinearities do not cause the two WIs to deviate substantially. The lowest is for the common carotid; this is mainly caused by a lower correlation for the 20–29 year age group, and the correlation is still strong for all other age groups.

The mean reflection coefficients in the common carotid and radial arteries show a relatively large discrepancy – they are higher for the noninvasive WI in all instances. For the common carotid, the magnitude for noninvasive WI is approximately constant across age groups, whereas the invasive WI is of smaller magnitude in the 20–29 year age group and grows with increasing age, narrowing but not closing the gap. For the radial, both of the reflection coefficients follow the same trend, but that trend is neither a consistent increase nor decrease with increasing age. For the brachial artery and thoracic aorta, there is close agreement for all age groups. The error in the loop-derived PWV causes the reflection coefficient to decrease significantly in the carotid in both the invasive and noninvasive cases; Figure 4 helps to elucidate the mechanism behind this – the poor separation achieved in the carotid leads to a more significant drop in the R wave than the S wave. For the other arteries, the reflection coefficients are similar regardless of the PWV used.

The mean systolic wave energy and peak ratios show excellent agreement for all age groups and arteries and regardless of whether the theoretical or loop-derived PWV was used, implying that they could both be robust metrics when using noninvasive WI as an indicator of heart performance. For both ratios, there is a high error when considering all subjects together, but this mainly reflects the variation between age groups: for both the invasive and noninvasive WI, the systolic wave energy ratios grow between the 20–29 and 40–49 year age groups for all arteries and then have small, inconsistent trends thereafter. There is also a greater variation between subjects for the younger groups. There is a slight error when using the loop-derived PWV to calculate the peak ratio in the carotid, but it is well within acceptable limits.

Both the mean start and peak delays in the common carotid and the radial arteries show disparity between formulations, though not in a consistent way. In the carotid, both delays start relatively large and fall for the noninvasive formulation and start relatively small and grow for the invasive formulation, eroding the gap by the oldest age groups. For the radial, the peak delays approximately match between formulations, but the start delays do not, with no appreciable trend for the invasive formulation but with a drop with increasing age for the noninvasive formulation. Again, the high error when considering all subjects reflects the variation between age groups, with the errors much smaller when observing the age-stratified data. The loop methods introduced significant discrepancies in a few cases: with the invasive start and peak delays in the carotid, the trends are completely reversed, with both instead starting at a much larger value and decreasing with age; with the invasive start delay in the radial artery, the magnitude is increased for all ages and decreases with age, as opposed to having no appreciable trend.

## Discussion

Overall, there was good agreement between the invasive and noninvasive formulations of WI (Figure 9). The biggest differences occurred in the common carotid and radial arteries; both of these arteries were modelled with large wall viscosities. The brachial was modelled to have a larger degree of strain-stiffening than the carotid because of its smaller diameter, yet did not display similar differences between the two WI formulations. This provides evidence that viscoelasticity is the dominant nonlinearity, so noninvasive WI will most closely resemble invasive WI in arteries that have low levels of this property rather than strain-stiffening.

The increase in arterial diameter with increasing age causes a decrease in both viscoelasticity and strain-stiffening as modelled by equations (5), (6) and (10). Hence, the older the subject, the smaller the differences between the two formulations. This trend is most visible for the metrics that differed the most between formulations – the reflection coefficients and the delays – and was strongest in the carotid. This is relevant clinically, as the prevalence of cardiovascular disease correlates with age;^35^ if the older patients show the closest agreement between the two WIs, that supports the equivalence of noninvasive WI as a means of diagnosis.

As noted above, reflection coefficients in the carotid and radial arteries show different trends with age: in the carotid, the noninvasive reflection coefficient is approximately constant and the invasive reflection coefficient increases with age; in the radial, both reflection coefficients show the same inconsistent trend. A likely contributing factor is the structural relationship of the parent artery and the two daughter arteries: the common carotid was modelled as elastic, bifurcating into two muscular arteries, whereas the radial and its daughter arteries are all muscular. The reflection coefficient at a junction, as modelled by equation (14), is dependent on both PWV and area. As we progress through the age groups, the multipliers given in Table 2 result in an increasing mismatch between elastic and muscular arteries; this acts to increase the reflection coefficient at the carotid bifurcation but does not have the same effect at the radial bifurcation.

Reflection coefficients obtained with the noninvasive formulation are consistently higher than those obtained with the invasive formulation in the carotid and radial arteries; there is a greater relative magnitude of the R wave. The reason for this is unclear. One explanation could stem from the fact that viscoelasticity causes the change in diameter to lag behind the change in pressure; as the reflected R wave returns to the measurement site after peak systole, although both diameter and pressure are falling, the diameter may be falling faster, giving a higher WI for the noninvasive formulation. Regardless of the mechanism causing the discrepancy, it is important that the ratio of the peak magnitudes of the R and S waves be generally greater for noninvasive WI.

Arteries stiffen as age increases, which leads to an increase in PWV and the expectation of an earlier arrival of reflected waves. This was observed in all cases other than for both delays in the carotid and the start delay in the radial, all three for the invasive formulation. Generally, the delay magnitudes matched quite well between formulations, though not in all cases; they differed significantly when the loop methods were used. Further investigation of the mechanisms behind these anomalies is required.

The systolic wave energy ratio and peak ratio were the most robust measures, with excellent agreement between invasive and noninvasive WI across all ages and regardless of the method used to calculate the PWV. While the nonlinear behaviour of the arterial wall does result in differences between the two formulations, using ratios appears to eliminate the majority of these differences. In the case of the systolic wave energy ratio, this is fairly intuitive. The S wave area contributes to the denominator as well as the numerator; this will damp the influence of any change in its area caused by switching from the invasive to the noninvasive formulation. With the peak ratio, although the S and D waves elicit opposite responses – the former causing an increase in velocity, pressure and diameter and the latter a decrease – viscoelasticity, the dominant nonlinearity, causes diameter changes to lag behind pressure changes for both. We therefore expect similar changes between formulations for both of these waves, again reducing the impact of the formulation on the ratio. Due to the robustness of these ratios, should a future study show a correlation between either of them and heart dysfunction or other cardiovascular pathology, it is reasonable to conclude that noninvasive WI has an equivalent diagnostic value to invasive WI.

Determining PWV from the loop methods leads to moderate or significant changes in all metrics other than the systolic wave energy ratio and the peak ratio in both the carotid and radial arteries; therefore, they should be used with caution. Nevertheless, noninvasive WI maintains its potential for introduction into the clinic: the PWV is needed to separate the intensities of forward and backward travelling waves but is not necessary for the analysis of total WI, and as noted, the systolic wave energy and peak ratios remain robust metrics. Note also, that differences between the invasive and noninvasive formulations do not necessarily imply that the former is superior, even though it entered practice first. Only their diagnostic and prognostic utility can establish which method is superior.

Finally, it is important to consider the sampling frequency. The one used in this study equates to an ultrasound frame rate of 1000 frames per second. If B-mode is used to measure velocity by ultrasound image velocimetry (UIV), a high frame rate is necessary to accurately resolve the velocity waveform, particularly at times of high gradient such as early systole. As the calculation of WI and the loop methods that estimate PWV are based on gradients, inaccuracies due to insufficient temporal resolution will affect the wave separation and WI metrics. When using UIV, the necessary frame rate is only achievable with ultrafast ultrasound devices;^36^ conventional scanners would not have the capacity to measure the velocity waveform with the same fidelity, so can only produce a downsampled version of the WIs shown here. MRI does not rely on velocimetry and has previously been used in several studies to obtain noninvasive WI with temporal resolutions of around 10 ms, either through calculating conventional WI through estimation of the blood pressure waveform^37^ or a noninvasive form of WI based on area or diameter,^38–41^ but the relatively high expense and low availability of MRI currently prohibit its widespread use.

### Limitations

1. Despite the accuracy and ease of 1D computational modelling, the results are only as valid as the assumptions, equations and estimations used; experimental studies are required to validate the conclusions drawn here.
2. There is a lack of physiological data for some of the parameters used, particularly the strain-stiffening parameter. Accuracy could be increased with further experimental data concerning how PWV behaves with distending pressure.^23^
3. The age stratification is somewhat arbitrary: binning ages into groups means that a 20 year old and a 29 year old are assumed to have similar arterial properties, but a 29 year old and a 30 year old different ones. Reasonable inferences can still be made about the separate groups and the population as a whole, however.

## Figures and Tables

**Figure 12.**
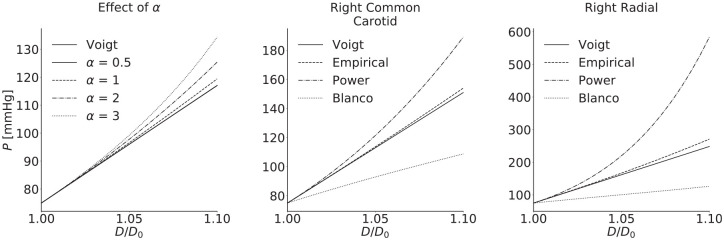
The effect of increasing α on the pressure–diameter relationship for the ascending aorta (left), together with the pressure–diameter relationships for the right common carotid (middle) and right radial (right) arteries, with the wave speeds of the separate tube laws scaled to match at the initial pressure. Note that the case α=0.5 gives an almost identical relationship to the Voigt-type law as expected.

## Appendix 1

### The empirical tube law

Following Parker,^16^ equation (4) may be rearranged to

(15)
$$\int_{A^{*}}^{A}\frac{dA\prime }{A\prime }=\int_{P^{*}}^{P}\frac{dP\prime }{\rho c^{2}}$$

which for the exponential law equation (3), is

(16)
$$\int_{A^{*}}^{A}\frac{dA\prime }{A\prime }=\int_{P^{*}}^{P}\exp\left[-\frac{\alpha }{\rho c^{{*}^{2}}}(P\prime -P^{*})\right]\frac{dP\prime }{\rho c^{{*}^{2}}}$$

which evaluates to

(17)
$$P=P^{*}-\frac{\rho c^{{*}^{2}}}{\alpha }\ln\left[1-\alpha \ln\left(\frac{A}{A^{*}}\right)\right]$$

A sensible choice of reference PWV is that of

(18)
$$c^{*}=\sqrt{\frac{\beta }{2\rho }}A^{{*}^{1/4}}$$

which gives

(19)
$$P=P^{*}-\frac{\beta \sqrt{A^{*}}}{2\alpha }\ln\left[1-\alpha \ln\left(\frac{A}{A^{*}}\right)\right]$$

Equation (18) is derived by applying equation (4) to the common elastic tube law

(20)
$$P=P^{*}+\beta \left(\sqrt{A}-\sqrt{A^{*}}\right)$$

and so such a choice ensures that the PWVs of the two laws match at the chosen reference area, and allows the law to be expressed in familiar variables of 1D computational blood flow modelling. A Taylor expansion about $A^{*}$ for both equations (19) and (20) allows further insight into the relationship between the two

(21)
$$\begin{array}{c} P\;\sim \;P^{*}+\frac{\beta }{2\sqrt{A^{*}}}\left(A-A^{*}\right)-\\ \frac{\beta }{4A^{{*}^{3/2}}}(1-\alpha )(A-A^{*}{)}^{2}+O[(A-A^{*}{)}^{3}] \end{array}$$

(22)
$$\begin{array}{c} P\;\sim \;P^{*}+\frac{\beta }{2\sqrt{A^{*}}}(A-A^{*})-\\ \frac{\beta }{8A^{{*}^{3/2}}}(A-A^{*}{)}^{2}+O[(A-A^{*}{)}^{3}] \end{array}$$

Notably, they are always equivalent in the linear regime and are equivalent in the quadratic regime for the choice α=0.5 . Essentially, such a choice models behaviour absent of strain-stiffening.

#### Computational formulae

One advantage of the tube law equation (19) is the ease of its computational implementation, owing to analytical formulae for the necessary components of the method of characteristics.^8,11^ The PWV and characteristics are

(23)
$$c=\sqrt{\frac{\beta }{2\rho \kappa }}A^{{*}^{1/4}}$$

(24)
$$W_{\pm }=U-U^{*}\pm \sqrt{\frac{2\beta }{\rho }}\frac{1-\sqrt{\kappa }}{\alpha }A^{{*}^{1/4}}$$

respectively, where

(25)
$$\kappa =1-\alpha \ln\left(\frac{A}{A^{*}}\right)$$

Furthermore, *A* can be calculated from the characteristics as

(26)
$$A=A^{*}\exp\left[\xi \left(2-\alpha \xi \right)\right]$$

where

(27)
$$\xi =\sqrt{\frac{\rho }{8\beta }}\frac{\left(W_{+}-W_{-}\right)}{A^{{*}^{1/4}}}$$

The other formulae are unchanged from the literature.

#### Justification

Other nonlinear tube laws exist, and this section details the reasoning behind the chosen law.

One nonlinear law is the power law^13^

(28)
$$P=P^{*}+\frac{2\rho c^{{*}^{2}}}{b}\left[{\left(\frac{A}{A^{*}}\right)}^{b/2}-1\right]+\frac{\Gamma }{\sqrt{A}}\frac{\partial A}{\partial t}$$

where *b* is a parameter which varies between each artery. There is a paucity of available empirical data for *b*, but it can be estimated using a collapse pressure $P_{\mathrm{collapse}}$

(29)
$$b=\frac{2\rho c^{{*}^{2}}}{P^{*}-P_{\mathrm{collapse}}}$$

For the arteries involved in this study, the estimated values were between 3 and 20. Due to the significant influence of *b* on the behaviour of the law and thus the large dependence on estimation, as well as the large variation of *b* between arteries, we experienced convergence issues with both the baseline model and generated subjects. Therefore, this law was not used.

Another law is the one used by Blanco et al.^42^

(30)
$$P=P^{*}+\frac{\pi hR^{*}}{A}\left[E_{E}\varepsilon +E_{C}\epsilon_{r}\ln(e^{u}+1)+K_{M}\overset{\cdot }{\varepsilon }\right]$$

where

(31)
$$\varepsilon =\sqrt{\frac{A}{A^{*}}}-1,\;u=\frac{\varepsilon -\varepsilon_{0}}{\epsilon_{r}}$$

and $E_{E}$ and $E_{C}$ are the effective elastic moduli of elastin and collagen, respectively; $\varepsilon_{0}$ and $\epsilon_{r}$ characterise the recruitment of the different fibres; and $K_{M}$ is the effective viscoelastic parameter. The effective parameters are calculated by a rule of mixtures approach

(32)
$$E_{E}=W_{E}E_{e},\;E_{C}=W_{C}E_{c},\;K_{M}=W_{M}K_{m}$$

where $E_{e}$ and $E_{c}$ are the actual elastic moduli of elastin and collagen, respectively; $K_{m}$ is the wall viscosity of smooth muscle; and $W_{E},W_{C}$ and $W_{M}$ represent the corresponding proportion of the wall constituents and satisfy $W_{E}+W_{C}+W_{M}=1$ . This methodology requires knowledge of the wall composition of each artery; Blanco et al.^42^ obviate this requirement by dividing arteries into three groups with differing constituent proportions based on arterial radii. As this method does not take into account the variation beyond these three groups, and due to the lack of available data on wall composition for each artery, this law was also not used.

The Voigt-type viscoelastic tube law

(33)
$$P=P^{*}+\beta \left(\sqrt{A}-\sqrt{A^{*}}\right)+\frac{\Gamma }{\sqrt{A}}\frac{\partial A}{\partial t}$$

has been shown to give physiological results.^8^ The chosen reference PWV causes the empirical law equation (5) to closely match the behaviour of this already established law but improves upon it through the addition of strain-stiffening, with equation (10) resulting in the magnitude of this effect varying between arteries.

In this study, the α values were estimated to be between 0.5 and 3; pressure–diameter relations giving an indication of the effect of varying the magnitude of alpha, along with a comparison between the four laws for two of the arteries examined in this study, are given in Figure 12. For both the carotid and radial arteries, the empirical law results in an improvement over the Voigt-type law through modelling a small but significant amount of strain-stiffening; however, there is a lack of experimental data on arterial pressure-diameter relations, so it remains to be established in vivo which tube law is the most accurate.
